# Targeting Sphingolipid Metabolism as a Therapeutic Strategy in Cancer Treatment

**DOI:** 10.3390/cancers14092183

**Published:** 2022-04-27

**Authors:** Alhaji H. Janneh, Besim Ogretmen

**Affiliations:** Hollings Cancer Center, Department of Biochemistry and Molecular Biology, Medical University of South Carolina, Charleston, SC 29425, USA; janneh@musc.edu

**Keywords:** cancer, sphingolipids, sphingosine-1-phosphate (S1P), cell growth, ceramide, apoptosis, therapeutics

## Abstract

**Simple Summary:**

Sphingolipids, which are important cell membrane components, have critical roles in regulating cancer cell signaling to control pro-tumoral or antitumoral functions. Ceramide, which is the central sphingolipid, facilitates cancer cell death, while sphingosine-1-phosphate (S1P) induces tumor growth/metastasis and confers resistance to chemo-, immuno-, or radiotherapies. The aim of this review is to highlight the mechanistic strategies of targeting sphingolipid metabolism for cancer therapeutics.

**Abstract:**

Sphingolipids are bioactive molecules that have key roles in regulating tumor cell death and survival through, in part, the functional roles of ceramide accumulation and sphingosine-1-phosphate (S1P) production, respectively. Mechanistic studies using cell lines, mouse models, or human tumors have revealed crucial roles of sphingolipid metabolic signaling in regulating tumor progression in response to anticancer therapy. Specifically, studies to understand ceramide and S1P production pathways with their downstream targets have provided novel therapeutic strategies for cancer treatment. In this review, we present recent evidence of the critical roles of sphingolipids and their metabolic enzymes in regulating tumor progression via mechanisms involving cell death or survival. The roles of S1P in enabling tumor growth/metastasis and conferring cancer resistance to existing therapeutics are also highlighted. Additionally, using the publicly available transcriptomic database, we assess the prognostic values of key sphingolipid enzymes on the overall survival of patients with different malignancies and present studies that highlight their clinical implications for anticancer treatment.

## 1. Introduction

Sphingolipids are a class of interconvertible bioactive lipids that were first discovered in the 19th century and named after the Sphinx (a Greek mythological creature) because of the mysterious nature of their biochemical properties, having alcohol sphingosine as the common backbone [1,2,3]. Sphingolipids, such as ceramide, ceramide 1-phosphate (C1P), glucosylceramide, sphingosine, and sphingosine-1-phosphate (S1P), have long been implicated in different biological processes including cell migration, proliferation, and cell death [4,5,6,7,8]. Studies over the years have highlighted the significance of sphingolipids in human diseases [9] including but not limited to lysosomal storage diseases, autoimmune diseases, cardiovascular diseases, infectious diseases, inflammation [7,10], and cancer [8,11]. The dysregulation of sphingolipid metabolism in various human cancer types suggests that bioactive sphingolipids are vital for tumor growth and survival. Therefore, developing the most effective cancer therapeutic treatments may require the regulation and balancing of sphingolipid metabolic pathways.

Over the past few decades, there has been significant progress in elucidating the therapeutic roles of sphingolipids in cancer treatment. However, the potential benefits of targeting the sphingolipid metabolic pathway for cancer therapy have yet to be fully realized. In this review, we discuss the mechanisms of sphingolipids in tumor control based on studies from both human cancers and mouse models that have led to the recent clinical advancements of sphingolipid regulation in cancer therapy. We also evaluated the prognostic roles of sphingolipid metabolic enzymes using publicly available data sets.

## 2. Sphingolipid Metabolism in Tumor Pathogenesis

**Figure 1:**
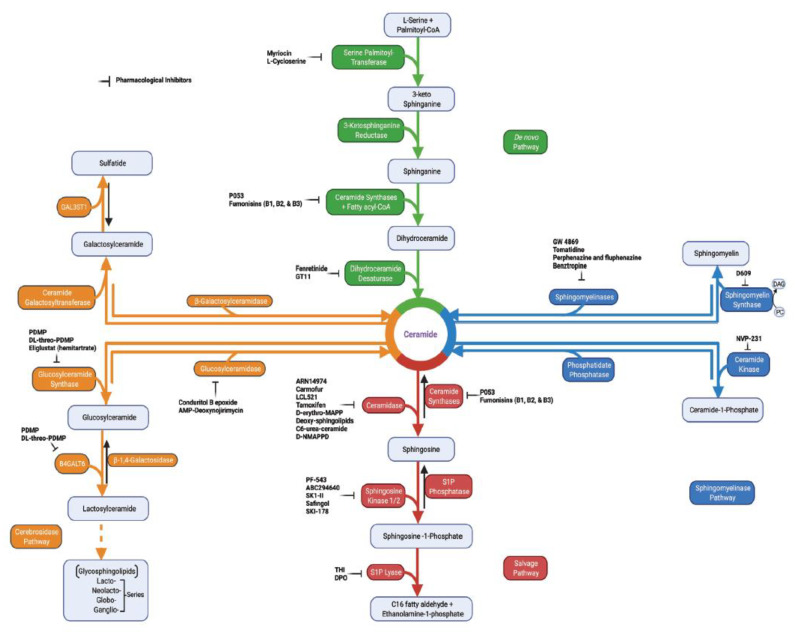
Sphingolipid metabolic pathways with selected inhibitors targeting enzymes. Ceramide, which is the intermediate molecule in sphingolipid metabolic pathway, can be formed either through de novo synthesis (green), sphingomyelin hydrolysis (blue), cerebrosides (orange), or salvage pathway (red). De novo synthesis starts with the functions of serine palmitoyltransferase (generates 3-keto sphinganine), 3-ketosphinganine reductase (generates sphinganine), (dihydro)ceramide synthases (generates dihydroceramide), and dihydroceramide desaturase (generates ceramide). The hydrolysis of sphingomyelin by the functions of sphingomyelinases can also generate ceramide (blue). Glucosylceramidase and β-galactosylceramidase can break down glucosylceramide and galactosylceramide, respectively, to generate ceramide (orange path). In the salvage pathway, ceramide synthases again can convert sphingosine to ceramide. In reverse, ceramide can be metabolized by ceramidases to generate sphingosine, which can then be phosphorylated to produce sphingosine-1-phosphate (S1P) by the functions of sphingosine kinases. S1P is broken down by the actions of S1P phosphatase to restore sphingosine or by S1P lyase functions, yielding ethanolamine 1-phosphate and C16 fatty aldehyde to exit the sphingolipid metabolic pathway. Sphingomyelin synthase transfers phosphorylcholine to ceramide from phosphatidylcholine (PC) to generate sphingomyelin and, thus, releasing diacylglycerol (DAG) [8]. Additionally, ceramide kinase functions to converts ceramide into ceramide-1-phosphate, while phosphatidate phosphatase functions to restore ceramide from ceramide-1-phosphate. In the generation of complex sphingolipids from ceramide, glucosylceramide synthase and ceramide galactosyltransferase produce glucosylceramide and galactosylceramide, respectively. Generation of glycosphingolipid series requires the synthesis of lactosylceramide from glucosylceramide (orange, dotted arrows). The enzymes can be inhibited by pharmacological inhibitors to regulate the sphingolipid metabolic pathway in both in vivo and in vitro studies. B4GALT6, beta-1,4-galactosyltransferase 6 [14]; GAL3ST1, galactosylceramide sulfotransferase; PDMP, 1-phenyl-2-decanoylamino-3-morpholino-1-propanol [15]; THI, 2-acetyl-5-tetrahydroxybutyl imidazole; DPO, 4-deoxy pyridoxine.

**Figure 2:**
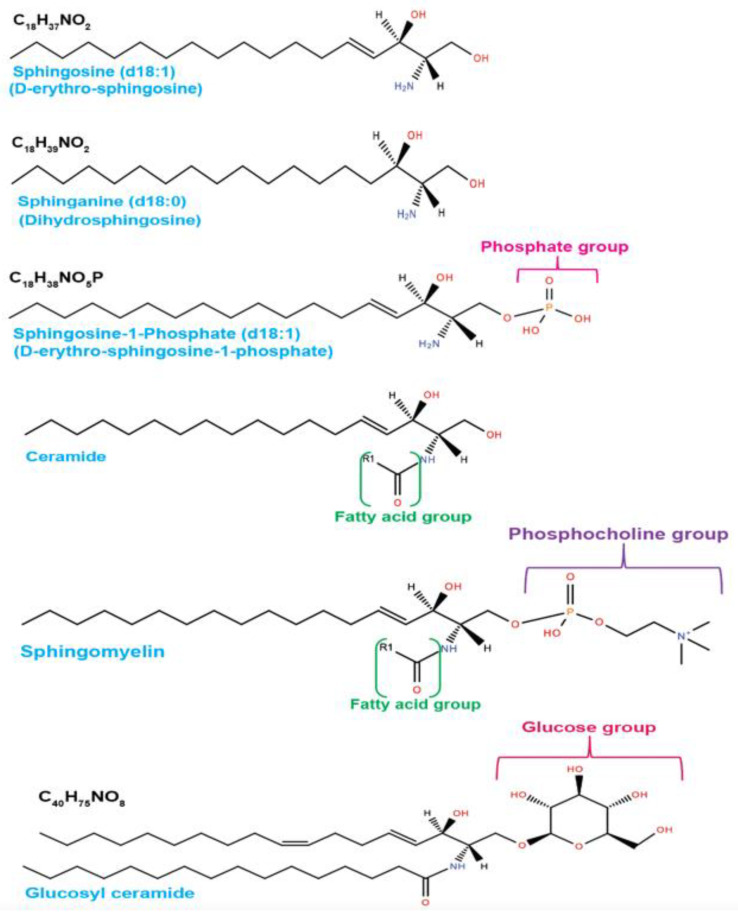
Sphingolipid structures.

In sphingolipid metabolism, the activation of de novo synthesis, sphingomyelinase, cerebrosidase, or the salvage pathways to generate ceramide and sphingosine (Figure 1) facilitates tumor cell death in response to cellular stress [8,12]. Ceramide, which is the central sphingolipid molecule, is composed of a sphingosine backbone and fatty acyl chain with variable carbon numbers (Figure 2). Interestingly, tumors with elevated levels of sphingolipid metabolic enzymes, such as sphingosine kinases, ceramide kinase, or acid ceramidase, generate crucial sphingolipids for pro-survival signaling functions [6,8]. The identification of the key enzymes in sphingolipid metabolic pathways over the years has paved the way for mechanistic investigations of the critical roles of sphingolipids in cancer progression. Moreover, there are myriads of available pharmacological inhibitors targeting sphingolipid enzymes (Figure 1) that can now be used to investigate the roles of sphingolipid metabolism in cancer pathogenesis, since many tumors have been shown to express an altered level of these sphingolipid enzymes [6,8,13].

Sphingosine(d18:1) and its derivative sphinganine(d18:0) forms the backbone of all sphingolipids. Sphingosine-1-phosphate, which is a bioactive sphingolipid, consists of a phosphate group that is attached to a sphingosine backbone at the first carbon (C1). Similarly, ceramide is composed of sphingosine backbone with 18 carbons and fatty acyl chain with variable carbon numbers (C14 to C26). Furthermore, ceramides can function as structural precursors for other sphingolipids such as sphingomyelin with a phosphocholine group and glucosylceramide with a glucose moiety. Structures were generated from https://www.lipidmaps.org (accessed on 22 April 2022).

## 3. Key Sphingolipid Enzymes and Their Roles in Cancer Progression

Most of the metabolic end-products in the sphingolipid pathway are regulated mainly by enzymes that are druggable (Figure 1 and Figure 2) and, thus, provide novel therapeutic targets for cancer treatment.

### 3.1. Sphingosine Kinases (SPHKs)

SPHKs are enzymes that catalyze the formation of S1P from sphingosine. Currently, there are two known SPHK isoenzymes that have been cloned and characterized—namely, SPHK1 and SPHK2. Both isoenzymes belong to the diacylglycerol kinase family and contain five (i.e., C1–C5) conserved domains [9,16]. SPHK1 is mainly localized in the cytosol with some intracellular organelle associations, while SPHK2 is found in nucleus, cytoplasm, and mitochondria [8,9,17]. Because of their crucial roles in sphingolipid metabolism, SPHK1 and SPHK2 both determine cell fates by regulating the balance between survival and cell death via S1P and ceramide metabolism.

Overexpression of SPHK1 in cancer cells has been shown to correlate with poor survival outcome for metastatic melanoma patients treated with an immune checkpoint inhibitor such as anti-PD-1 [18]. Interestingly, decreasing SPHK1 expression improves the efficacy of immune checkpoint inhibitors (anti-CTLA-4 and anti-PD-1 therapy) in melanoma, breast, and colon cancer mouse models [18]. Additionally, inhibiting SPHK1 enhances the metabolic activities of T cells and improves their antitumor functions against murine melanoma [19]. Moreover, anti-PD-1 and PF-543 (SPHK1 inhibitor) combinations improve the control of melanoma tumor growth [19]. SPHK1 has also been shown to play a crucial role in adipocyte-induced epithelial ovarian cancer (EOC). Adipocytes activate SPHK1 via S1PR1/3 and ERK phosphorylation to stimulate growth-promoting action leading to EOC cell proliferation [20]. SPHK1 upregulation promotes chronic inflammation and colitis-associated cancer development [21].

Similarly, increased SPHK2 expression levels correlate with augmented dihydropyrimidine dehydrogenase (DPD) in human colorectal cancer (CRC), and the inhibition of SPHK2 by SLR080811 effectively inhibits DPD expression and reverses 5-fluorouracil (5-FU) resistance in colorectal tumors of villin-Sphk2 Tg mice while also decreasing nuclear S1P concentration in tissues [22]. The colorectal tumors that were developed in Sphk2^−/−^ mice showed great sensitivity to 5-FU therapy, indicating that high SPHK2 expression in colorectal tumors yields resistance to 5-FU chemotherapy treatment [22].

Both SPHK1 and SPHK2 have been shown to be equally responsible for follicle-stimulating hormone (FSH)-induced cell proliferation of epithelial ovarian cancer. FSH induces the phosphorylation of both SPHK1 and SPHK2 enzymes to regulate the survival and growth of ovarian cancer cells via the ERK1/2 pathway [23].

Collectively, these data suggest that both SPHK1 and SPHK2 play crucial roles in stimulating tumor growth and survival, supporting the importance of regulating S1P generation for cancer treatment.

### 3.2. Sphingosine-1-Phosphate Lyase 1 (SGPL1)

SGPL1 is an enzyme localized in the endoplasmic reticulum that irreversibly breaks down S1P into C16 fatty aldehyde and ethanolamine-1-phosphate [24,25]. It provides the exit point for sphingolipid metabolism. SGPL1 knockout in mouse colon tissues (T-SGPL^−/−^) was shown to cause immediate and extensive colon tumor formation [26]. T-SGPL^−/−^ also stimulates cancer-induced inflammation and increased both S1P and sphingosine levels [26]. Additionally, SGPL1 knockout in mouse immune cells (I-SGPL^−/−^) also leads to S1P accumulation in the immune cells but causes delayed carcinogenesis compared to T-SGPL^−/−^ cells [26]. Another study has shown that a low probability of metastasis formation is associated with high native SGPL1 expression [24]. The native form of SGPL1 expression prevents S1P-induced migration and cell-colony formation of pediatric alveolar rhabdomyosarcoma (RMA) compared to SGPL1 mutant [24]. Moreover, silencing of SGPL1 influences the tumorigenic activity of established colorectal cancer cells and partial redifferentiation of colorectal cancer [27].

### 3.3. Ceramide Kinase (CERK)

CERK is an enzyme that catalyzes the phosphorylation of ceramide to form C1P, and its activity is known to be regulated by Ca^2+^ ions [28,29]. This enzyme was first discovered in synaptic vesicles from brain cells and has been shown to have a cytosolic localization and is found in the membrane fraction as well [29,30]. CERK overexpression has been shown to promote triple-negative breast cancer (TNBC) growth and migration and confer chemotherapy resistance to breast cancer cell lines [31]. Consequently, CERK siRNA knockdown improves TNBC chemotherapy efficacy and suppresses TNBC growth, migration, and survival [31]. Another study has shown that CERK expression in breast cancer cells promotes migration and invasion via the PI3K/Akt pathway [32]. Additionally, inhibiting CERK expression with either a pharmacological inhibitor (NVP-231) or genetic tools (shRNAs) significantly reduces the migratory potential and invasiveness in breast metastatic cell lines [32]. Similarly, NVP-231 induces programmed cell death by stimulating M phase cell cycle arrest in breast and lung cancer cell proliferation [33]. Moreover, Payne et al. [34] showed that CERK is needed for mammary tumor recurrence in murine breast cancer models, following HER2/neu pathway inhibition. Consistently, in human patients, the upregulation of CERK expression is associated with an elevated risk of breast cancer recurrence in women [34].

Taken together, these findings suggest that inhibiting CERK would provide a novel therapeutic target for cancer treatment.

### 3.4. Ceramidases (CDases)

CDases hydrolyze ceramide, by cleaving the fatty acid moiety from ceramide, to produce sphingosine. Currently, five human CDases have been cloned and are encoded by five distinct genes, categorized into three different classes—acid ceramidase (AC), neutral ceramidase (NC), and alkaline ceramidases 1–3 (ACER1–3) [35].

AC is a lysosomal ceramidase that is overexpressed in several cancer types [36,37]. For instance, AC was reported to regulate the switch between proliferative and invasive phenotype states in melanoma cells [38]. Using both cells isolated from human melanoma biopsies and melanoma cell lines, Leclerc et al. [38] showed that melanoma cells with proliferative activity displayed increased in ASAH1 expression. Consequently, the melanoma cells developed an invasive property after the loss of ASAH1, thus losing their proliferative phenotype and acquiring enhanced motile properties [38]. Interestingly, AC inhibition in colorectal cancer cell lines using pharmacological AC inhibitors (carmofur and LCL521) or siRNA knockdown of AC enhanced X-ray radiosensitivity by increasing apoptosis [39]. Correspondingly, patient-derived organoids with decreased AC expression showed more radiosensitivity compared to the patient-derived organoids with an elevated AC expression [39]. Similarly, AC deletion in melanoma cells enhanced doxorubicin-induced apoptosis [40]. Consistently, previous studies have also suggested a role of AC in chemo- and radiotherapy failures [36,41,42].

NC is localized in the plasma membrane, Golgi apparatus, and mitochondria [43]. Inhibition of NC in colorectal cancer induces a xenograft tumor growth delay [44]. Additionally, constitutively active AKT cells of xenograft tumors are resistant to NC inhibition [44]. Consistently, pharmacological inhibition (C6 urea–ceramide) and molecular inhibition (siRNA knockdown) of NC increases ceramide and decreases cell survival via elevated cellular apoptosis in colon cancer cells [45]. 

**Figure 3:**
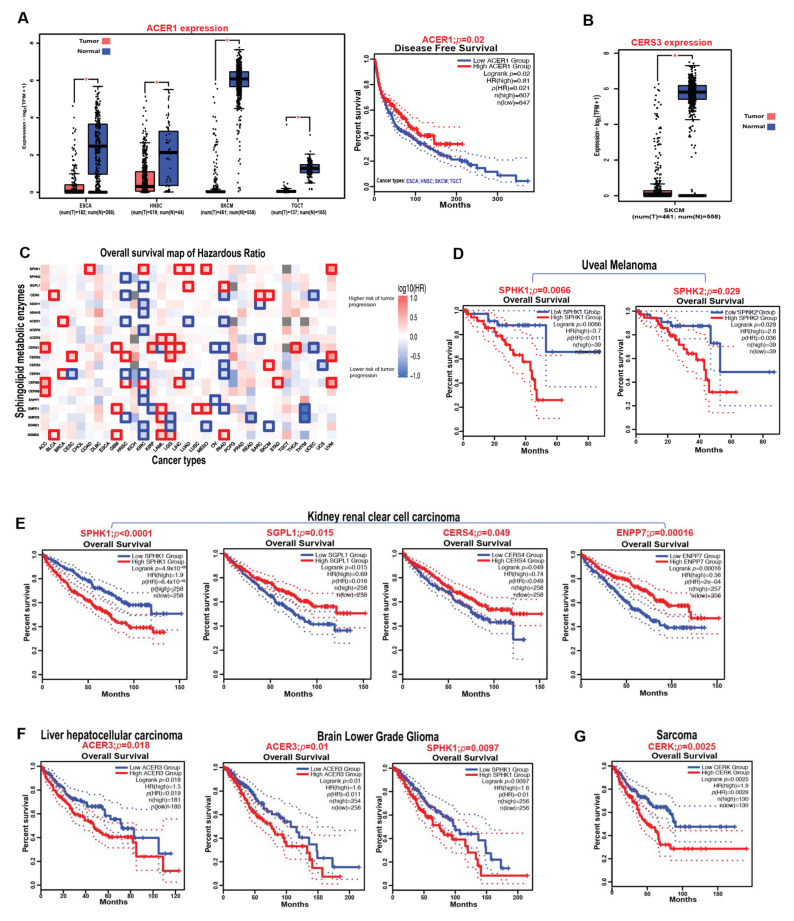
Expression effects of sphingolipid metabolic enzymes on the survival outcomes of cancer patients. (**A**) Box plots indicating the differential expression of ACER1 in ESCA, HNSC, SKCM, and TGCT patients compared to healthy controls. The Kaplan–Meier survival curve shows the overall survival impact of ACER1 expression in ESCA, HNSC, SKCM, and TGCT tumors combined. Tumor group, (T); normal group, (N). * *p*-Value < 0.05. The differential expression is calculated by the mean value of log2(TPM + 1). TPM, transcript per million. (**B**) Box plot indicating the differential expression of CERS3 in SKCM. Tumor group, (T); normal group, (N). * *p*-Value < 0.05. The differential expression was calculated by the mean value of log2(TPM + 1). TPM, transcript per million. (**C**) Heatmap representing the overall survival of hazardous ratios (HRs) predicting the risks of tumor progression in different malignancies based on the expression patterns of sphingolipid metabolic enzymes. The red colored blocks correspond to an increased risk of tumor progression when the enzyme is overexpressed, while the blue colored blocks correspond to a lower risk (protective function) when the enzyme is overexpressed. The bold outlined boxes indicate significance based on log-rank *p*  <  0.05. (**D**) Kaplan–Meier survival curves showing the overall survival impacts of SPHK1 and SPHK2 expressions in uveal melanoma. (**E**) Kaplan–Meier survival curves showing the overall survival impacts of SPHK1, SGPL1, CERS4, and ENPP7 expressions in kidney renal clear cell carcinoma. (**F**,**G**) Kaplan–Meier survival curves showing the overall survival impacts of ACER3 in liver hepatocellular carcinoma and brain lower-grade glioma: SPHK1 in brain lower-grade glioma (**F**) and CERK in sarcoma (**G**). Analysis was performed using the Gene Expression Profiling Interactive Analysis2 (GEPIA2) web server. ACC, adrenocortical carcinoma; BLCA, bladder urothelial carcinoma; BRCA, breast invasive; carcinoma; CESC, cervical squamous cell carcinoma and endocervical adenocarcinoma; CHOL, cholangiocarcinoma; COAD, colon adenocarcinoma; DLBC, lymphoid neoplasm diffuse large B-cell lymphoma; ESCA, esophageal carcinoma; GBM, glioblastoma multiforme; HNSC, head and neck squamous cell carcinoma; KICH, kidney chromophobe; KIRC, kidney renal clear cell carcinoma; KIRP, kidney renal papillary cell carcinoma; LAML, acute myeloid leukemia; LGG, brain lower-grade glioma; LIHC, liver hepatocellular carcinoma; LUAD, lung adenocarcinoma; LUSC, lung squamous cell carcinoma; MESO, mesothelioma; OV, ovarian serous cystadenocarcinoma; PAAD, pancreatic adenocarcinoma; PCPG, pheochromocytoma and paraganglioma; PRAD, prostate adenocarcinoma; READ, rectum adenocarcinoma; SARC, sarcoma; SKCM, skin cutaneous melanoma; STAD, stomach adenocarcinoma; TGCT, testicular germ cell tumors; THCA, thyroid carcinoma; THYM, thymoma; UCEC, uterine corpus endometrial carcinoma; UCS, uterine carcinosarcoma; UVM, uveal melanoma; SPHK1, sphingosine kinase 1; SPHK2, sphingosine kinase 2; SGPL1, sphingosine-1-phosphate lyase 1; CERK, ceramide kinase; ASAH1, acid ceramidase; ASAH2, neutral ceramidase; ACER1, alkaline ceramidase 1; ACER2, alkaline ceramidase 2; ACER3, alkaline ceramidase 3; CerS1, ceramide synthase 1; CerS2, ceramide synthase 2; CerS3, ceramide synthase 3; CerS4, ceramide synthase 4; CerS5, ceramide synthase 5; CerS6, ceramide synthase 6; ENPP7, alkaline sphingomyelinase; SMPD1, acid sphingomyelinase; SMPD3, neutral sphingomyelinase; SGMS1, sphingomyelin synthase 1; SGMS2, sphingomyelin synthase 2.

ACERs (i.e., ACER1, ACER2, and ACER3) are closely related family members with distinct biological functions in regulating sphingolipid metabolism [46]. ACER1 has been shown to play a crucial role in mammalian skin homeostasis [47], and it is found to be localized in the endoplasmic reticulum [43,48]. The role of ACER1 in cancer has not been elucidated and, therefore, ACER1-specific functions in cancer progression and survival are currently unknown. However, analysis from the publicly available transcriptomic database in The Cancer Genome Atlas (TCGA) indicates that ACER1 is significantly downregulated in human skin cutaneous melanoma, head and neck squamous cell carcinoma, testicular germ cell tumors, and esophageal carcinoma primary tumors compared to normal tissues. Meanwhile, given the low expression of ACER1, TCGA data combining patients with these four different cancer types altogether had worse prognostic outcome (Figure 3A). It is particularly important to investigate the specific functions of ACER1 in melanoma (i.e., skin cancer), since ACER1 is crucial for keratinocyte differentiation [48], and there is already a known existing crosstalk between melanocytes, keratinocytes, and melanoma [49]. It would be interesting to investigate whether ACER1 has a protective function in melanoma development. ACER2 is a Golgi ceramidase [50] that has a higher affinity towards unsaturated long-chain ceramides (C18:1, C20:1, and C24:1 ceramide) [51]. ACER2 was reported to be overexpressed in hepatocellular carcinoma (HCC) tissue and to induce growth, invasion, and migration in HCC cell lines via sphingomyelin phosphodiesterase acid-like 3B (SMPDL3B) [52]. In another study, ACER2 and sphingosine levels were shown to be upregulated in DNA-damaged tumor cells [53]. The ACER2/sphingosine upregulation pathway stimulates programmed cell death in a human colorectal carcinoma cell line (i.e., HCT116 cells) by increasing reactive oxygen species (ROS) production in response to DNA damage [53]. Additionally, ACER2 regulates p53-induced autophagy and apoptosis via sphingosine and ROS generation in human non-small cell lung carcinoma cell line (i.e., H1299 cells) [54]. ACER3 is localized in both the endoplasmic reticulum and Golgi complex, and it hydrolyzes unsaturated long-chain ceramides (C20:4, C20:1, and C18:1 ceramide), phytoceramides, and dihydroceramides to produce sphingosine [55,56]. Knockdown of ACER3 suppressed tumor growth and promoted apoptosis in HCC cells [57]. Similarly, ACER3 deficiency decreased acute myeloid leukemia (AML) cell growth and increased apoptosis in the AML cells via limiting AKT signaling [58]. Interestingly, ACER3 was shown to play a vital role in regulating the expression of pro-inflammatory cytokines of the innate immune system cells via C18:1 ceramide [59]. Additionally, ACER3 and C18:1 ceramide dysregulation contribute to the pathogenesis of cancer as an inflammatory disease [59].

### 3.5. Ceramide Synthases 1–6 (CerS1–6)

A total of six mammalian CerS enzymes (CerS1–CerS6) have been identified, cloned, and described [60,61]. Their general function is to catalyze de novo synthesis of ceramides [62,63], which can induce apoptosis [64]. CerS1–6 enzymes are also known as the longevity assurance homologue of yeast lag1 (Lass1) [12,61], and they have been implicated in the regulation of programmed cell death [65,66].

CerS1, the first identified mammalian CerS that catabolizes the synthesis of C18 ceramide [67,68], was shown to be downregulated in oral cancer tissues and cell lines [69]. The downregulation of CerS1 promotes the aggressiveness of oral squamous cell carcinoma and chemotherapy drug (cisplatin) resistance, while CerS1 overexpression induced sensitization to cisplatin via regulating cell death [69]. Similarly, histone deacetylase 1 (HDAC1) and microRNA-574-5p axis was found to repress CerS1 and alter C18 ceramide generation in head and neck squamous cell carcinoma (HNSCC), thereby allowing tumor growth and proliferation [70]. Thus, CerS1/C18 ceramide expression inhibits HNSCC xenograft growth and induces cell death [71,72,73]. Additionally, targeting Fms-like tyrosine kinase 3 (FLT3)–internal tandem duplication (ITD) induces mitophagy, leading to AML cell death via CerS1/C18-mediated mitophagy [74]. The CerS enzyme that synthesizes very-long-chain ceramides, CerS2, was reported to have an antimetastatic gene function in ovarian cancer cells [75]. Downregulation of CerS2 in ovarian cancer cell lines stimulates in vivo metastasis and invasiveness [75]. Moreover, CerS2 alternative splicing modulates cancer cell proliferation and migration in luminal B breast cancer [76]. Interestingly, overexpression of CerS2 and C24 ceramide generation in HeLa cells partially prevents programmed cell death induced by ionizing radiation [77]. Loss of CerS3, which catabolizes C24 and long acyl chain ceramides synthesis [68], was reported to cause lethality in skin barrier disturbance [78]. Although the specific functions of CerS3 in cancer is unknown, and data from TCGA database indicates that CerS3 expression is decreased in human skin cutaneous melanoma (Figure 3B). CerS4, which catabolizes the synthesis of C18–C20 ceramides, regulates cancer cell migration and invasion [79]. Knockdown of CerS4 increases migration in A549 cells, and the restoration of CerS4 generates C18–C20 ceramides to inhibit cancer cell migration and invasion [79]. Although both CerS5 and CerS6 generate C16 ceramide, only CerS6 appears to regulate C16 ceramide in mitochondria and mitochondria-associated membranes [80]. Consequently, in activated aging T cells, the C14/C16 ceramides generated by CerS6 stimulate mitophagy and attenuate the T cells’ antitumor functions [81]. Conversely, CerS5 knockout was shown to stimulate colon cancer development in azoxymethane (AOM) and dextran sulfate sodium (DSS) colitis-associated colon cancer models [82]. Interestingly, CerS6 overexpression and C16 ceramide generation promotes cell proliferation, colony formation, and invasion via the AKT1/FOXP3 pathway in pancreatic ductal carcinoma (PDAC) cell lines [83], consistent with its proliferative roles in head and neck squamous cell carcinoma and lung cancer cell lines [71,84]. Additionally, high CerS6 expression levels predicted worse prognosis in PDAC patients and was positively correlated with disease progression [83]. Moreover, for its antiproliferative and pro-apoptotic roles, CerS6 expression was shown to increase p53 protein half-life via a positive feedback loop in polyploid giant cancer cells (PGCCs) [85]. In addition, CerS6 and p53 co-expression nullified the ability of PGCC to form offspring with a high proliferative and therapy-resistant phenotype [85]. These studies suggest that although ceramide accumulation mainly induces cancer cell death and antiproliferative signaling in many tumors, it might also have proliferative functions depending on the downstream signaling targets.

### 3.6. Sphingomyelinases (SMases)

SMases, or sphingomyelin phosphodiesterases, catalyze the conversion of sphingomyelin to ceramide and phosphocholine [86]. SMases are classified into three groups based on their optimum pH (i.e., alkaline, acid, and neutral) [86].

Alkaline SMase (Alk-SMase or ENPP7), which is found in intestinal mucosa, bile, and liver, was shown to reduce colon cancer progression in a mice model [87]. Alk-SMase knockout mice showed a decrease in ceramides, increased S1P levels, and resulted in enhanced colonic tumorigenesis induced by AOM/DSS treatment [87]. Acid SMase (ASMase or SMPD1) is an enzyme found in lysosomes, and its deficiency leads to an inherited lysosomal disease [88]. Interestingly, adult patients with chronic visceral (CV) ASMase deficiency (CV-ASMD) were observed to have an abnormally elevated incidence of cancers [88]. Thus, the risk of cancer was shown to be associated with CV-ASMD disease severity [88]. Furthermore, ASMase induction [89] in platelets induces B16F10 melanoma metastasis, consequently inhibiting ASMases with amitriptyline-prevented tumor metastasis by 75% [90,91]. In addition, the downregulation of neutral SMase 2 (NSMase2, also known as SMPD3) contributes to melanoma immune escape to enhance tumor progression, while the overexpression of wild-type nSMase2 enhances the efficacy of anti-PD-1 antibody therapy in both melanoma and breast cancer mouse models [92]. Moreover, SMPD3 downregulation promotes tumor progression in oral squamous cell carcinoma (OSCC) [93].

### 3.7. Sphingomyelin Synthase (SMS)

There are two known isoforms of SMS—SMS1 and SMS2. Both isoforms catalyze the same reaction to produce sphingomyelin and diacylglycerol [94,95]. SMS1 is localized in the trans-Golgi apparatus, while SMS2 is mainly localized in the plasma membranes [94,95]. Although the specific roles of SMS1 in cancer cell growth and survival remains to be elucidated, SMS2 has been shown to have pro-tumoral [96,97] or apoptotic [98] functions in a cancer-type dependent manner, signifying the need for further studies of SMS-specific roles in tumor control. However, it was recently shown that SMS2 but not SMS1 was upregulated in ovarian cancer tissues and cell lines and, consequently, SMS2 overexpression promoted cancer cell growth and migration [99], suggesting a therapeutic function of SMS2 inhibition in ovarian cancer treatment.

Collectively, targeting these sphingolipid enzymes while monitoring and controlling sphingolipid accumulations would be important for effective cancer therapy, since the altered expression of these enzymes regulate tumor growth/survival and cell death (Table 1).

### 3.8. Prognostic Impact of Sphingolipid Metabolic Enzymes on the Survival of Cancer Patients

Using the GEPIA2 (Gene Expression Profiling Interactive Analysis, version 2) web server with TCGA data sets [100,101], we assessed the effects of key sphingolipid enzymes on the overall survival of patients with different types of cancer. The increased expression of these metabolic enzymes was observed to be associated with higher or lower risks of tumor progression depending on the cancer type, as indicated by the red blocks (poor prognosis) or blue blocks (good prognosis) on the heatmap, respectively (Figure 3C). For instance, SPHK1 overexpression is significantly associated with higher risks of tumor progression or worse prognostic outcome in colon adenocarcinoma (COAD), kidney renal clear cell carcinoma (KIRC), liver hepatocellular carcinoma (LIHC), lung adenocarcinoma (LUAD), mesothelioma (MESO), and uveal melanoma (UVM) as indicated by the red bold outlines on the heatmap (Figure 3C). Correspondingly, CERS4 overexpression is significantly associated with lower risks of tumor progression or better prognostic outcome in cervical squamous cell carcinoma and endocervical adenocarcinoma (CESC), head and neck squamous cell carcinoma (HNSC), KIRC, LUAD, and pancreatic adenocarcinoma (PAAD) as indicated by the blue bold outlines on the heatmap (Figure 3C). Specifically, in uveal melanoma, overexpression of both SPHK1 and SPHK2—the enzymes that catalyze the production of S1P—is associated with worse or unfavorable disease outcomes compared to the low-expression groups (Figure 3D). Similarly, in kidney renal clear cell carcinoma, worse or unfavorable prognosis is associated with high expression of SPHK1, low expression of SGPL1, CERS4, and ENPP7 (Alk-SMase) (Figure 3E), possibly due to the sustained production of S1P as indicated by high SPHK1 with low SGPL1 levels and a decrease in ceramide production to inhibit apoptosis as indicated by the low levels of CERS4 and ENPP7. Additionally, ACER3 expression, which has been shown to regulate cancer pathogenesis [57,58], was observed to have an unfavorable overall survival outcome in liver hepatocellular carcinoma and brain lower-grade glioma (Figure 3F). This observation was also consistent with the poor overall survival outcome associated with high SPHK1 expression in brain lower-grade glioma (Figure 3F). Importantly, CERK expression, which catalyzes the formation of C1P, was observed to be associated with worse overall survival in sarcoma (Figure 3G).

Overall, these observations suggest that sphingolipid metabolic enzymes play an important role in regulating tumor pathogenesis and would therefore provide therapeutic targets for cancer treatments in patients.

## 4. S1P Signaling in Cancer

S1P is a bioactive lipid mediator that acts as a cell signaling molecule to regulate various biological processes including cell survival, proliferation, and motility. S1P is generated intracellularly by sphingosine kinases (SPHK1/SPHK2), and the cytosolic S1P produced by SPHK1 is exported out of the cell and into the extracellular space via specific transporters, since S1P cannot freely cross the plasma membrane barrier due to the fact of its polar head group [102]. The S1P released in the extracellular space signals through S1P receptors (S1PRs) in a process called “inside-out” signaling [2], which has been shown to occur in many cancer types [102,103,104]. Although S1P produced by both SPHK1 and SPHK2 have intracellular functions [105,106,107], extracellular S1P mainly produced by SPHK1 play main roles in controlling “inside-out” signaling process [102].

### 4.1. S1P Transporters

**Figure 4:**
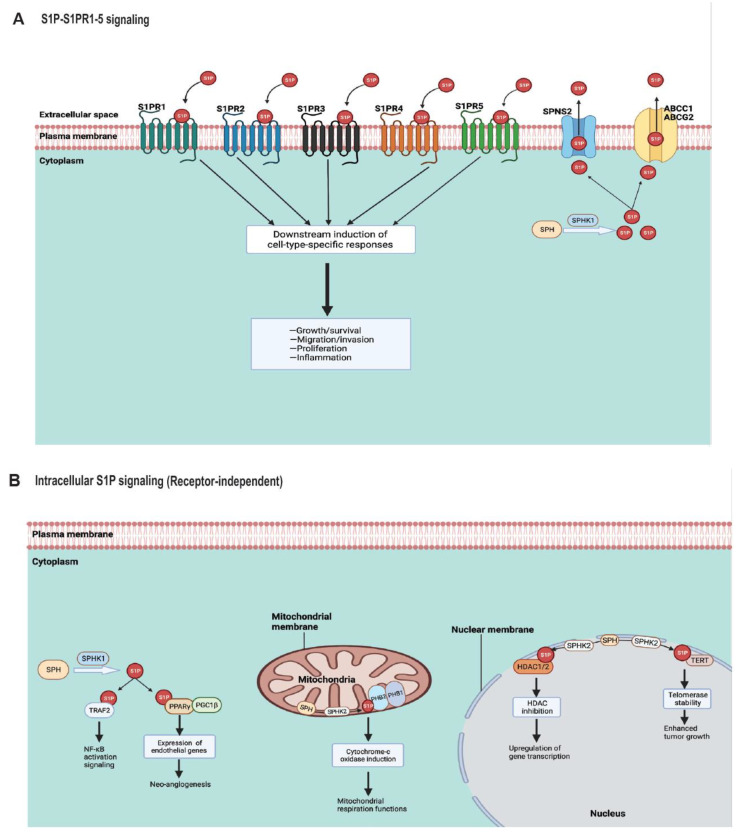
S1P receptor and receptor-independent signaling. (**A**) SPHK1 catalyzes the synthesis of S1P from SPH in the cytoplasm. S1P then exit the cytoplasm and into the extracellular space via SPNS2, ABCC1, or ABCG2 transporters. The secreted S1P can engage the five known S1P specific G protein-coupled receptors (S1PR1–5) for cellular signaling leading to a downstream induction of cell-type-specific responses to stimulate cell growth/survival, migration/invasion, proliferation, and/or inflammation. (**B**) S1P can also function independent of S1PRs. In the cytoplasm, SPHK1-generated S1P can bind TRAF2 at the N-terminal RING domain, leading to NF-κB signaling activation downstream. SPHK1-derived S1P can also bind and activate PPARγ, which then allows for the recruitment of PGC1β, to form the SlP/PPARγ/PGC1β complex, inducing PPARγ-dependent genes and neo-angiogenesis. SPHK2-generated S1P in the mitochondria can bind homomeric PHB2 without binding to PHB1 to induce cytochrome c oxidase or complex IV and mitochondria respiration functions. In the nucleus, SPHK2-derived S1P can bind HDAC1 and HDAC2, inhibiting their activities to stimulate the upregulation of gene transcriptions. Additionally, SPHK2-generated S1P can also bind TERT in the nuclear membrane to stabilize telomerase and enhance tumor growth. SPH, sphingosine; SPHK1, sphingosine kinase 1; SPHK2, sphingosine kinase 2; S1P, sphingosine-1-phosphate; SPNS2, protein spinster homolog 2; ABCC1, ATP-binding cassette sub-family C member 1; ABCG2, ATP-binding cassette sub-family G member 2; S1PR, sphingosine-1-phosphate receptor; TRAF2, TNF receptor-associated factor 2; NF-κB, nuclear factor-κB; PPARγ, peroxisome proliferator-activated receptor-γ; PGC1β, PPARγ co-activator 1β; PHB1, prohibitin 1; PHB2, prohibitin 2; HDAC1, histone deacetylase 1; HDAC2, histone deacetylase 2; TERT, telomerase reverse-transcriptase.

The S1P generated intracellularly is exported into the extracellular space leading to inside-out signaling in the tumor microenvironment via specific S1P transporters including protein spinster homologue 2 (SPNS2), major facilitator superfamily d2b (Mfsd2b), ATP-binding cassette sub-family C member 1 (ABCC1), and ATP-binding cassette sub-family G member 2 (ABCG2) (Figure 4A).

SPNS2 belongs to the major facilitator superfamily (MFS)—the largest secondary transporter protein family with 12 putative transmembrane protein domains. It has 504 amino acid residues in zebrafish but 549 amino acid residues in human and mouse [108]. The functional roles of SPNS2 as a transporter for S1P signaling were observed in zebrafish models, where it was shown that cardia bifida (split heart abnormality or defective heart development) occurs because of a point mutation in spns2 which that inhibits S1P signaling, causing migration defect of myocardial precursors [104,109,110]. Interestingly, this defect could be rescued by exogenous S1P addition [109]. Recent studies have also shown that other MFS family members, namely, MFS domain-containing 2a and 2b (Mfsd2a and Mfsd2b) play vital roles in transporting S1P alongside SPNS2. Mfsd2a and Spns2 form a protein complex that allows for effective/sufficient S1P export from endothelial cells in the brain [111]. Circulating S1P is transported via both Mfsd2b and SPNS2. In endothelial cells, SPNS2 is the major S1P transporter, while Mfsdb2 is the key S1P transporter in erythrocytes and platelets [112,113,114,115,116,117]. Remarkably, spns2 deletion in mice, whether globally or in a lymphatic endothelial-specific manner, leads to improved tumor killing and significantly decreased metastatic burden in Spns2^−/−^ mice as a result of increased levels of natural killer cells and effector T cells [118]. Similarly, SPNS2 has been shown to deliver S1P to S1PR2, leading to increased epidermal growth factor (EGF)-mediated cancer cell invasion [119], suggesting that SPNS2 inhibitors may be useful therapeutics for cancer treatment. Contrastingly, low SPNS2 levels are associated with worse clinical prognosis in colorectal cancer, while the ectopic expression of SPNS2 was shown to decrease migration, invasion, and metastasis in colorectal cancer cell lines via AKT signaling pathway [120]. These contrasting results could be explained by the need of SPNS2–S1P signaling to stimulate specific or opposing S1P receptors in a cancer type-specific manner.

ABCC1 and ABCG2 are ABC transporters that have been implicated in lipid signaling by transporting S1P across cell membranes [104]. ABCC1 [121] is another member of the ABC transporter family, multidrug resistance-associated protein 1 (MRP) shown to confer resistance to anticancer drugs [122,123]. ABCG2 is identified as an ABC transporter, a breast cancer resistance protein, that mediates S1P efflux from cells [123,124]. Both ABCC1 and ABCG2 were reported to mediate estradiol-induced S1P release in breast cancer cells [8,124]. Consequently, inhibiting ABCC1 or ABCG2 with pharmacological inhibitors decreases estradiol-mediated release of S1P and dihydro-S1P and ERK1/2 activation in breast cancer [124]. Additionally, decreasing ABCC1 increases ABCG2 expression levels and vice versa [124]. The presence of these ABC transporters in cancer cells may explain why most breast cancer types are resistant to chemotherapy treatment. Thus, understanding these transporters for S1P signaling could help understand cancer chemotherapy resistance.

### 4.2. S1P Receptors (S1PR1–5)

The released S1P into the extracellular space via its transporters can engage the five known S1P-specific G protein-coupled receptors (S1PR1–S1PR5) for cellular signaling (Figure 4A). This leads to context-dependent specific functions including the proliferation, migration, and growth/survival of cancer cells. In a physiological context, circulating S1P uses protein carriers to engage its receptors. For instance, S1P could either bind to high-density lipoprotein (HDL) through apolipoprotein M (ApoM), HDL/ApoM, or albumin [125,126]. In endothelial cells, evidence showed that S1PR1 signaling is more dependent on HDL-bound S1P compared to albumin-bound S1P for their downstream activation of Akt and eNOS [9,127], which are known targets for cancer treatment. Additionally, HDL-bound S1P increases S1PR1 expression levels while decreasing its degradation rate compared to albumin-bound S1P [127]. This result is consistent with the findings that low ApoM production in aged mice reduces S1P signaling via S1PR1 in lung and kidney endothelial cells leading to maladaptive repair and fibrosis compared to young mice [128]. Moreover, S1PR1, as a pro-tumorigenic factor, has been shown to activate cancer cell signaling pathways leading to invasion, migration, and proliferation [102]. Consistently, S1P–S1PR1 signaling in tumor cells or the tumor microenvironment induced persistent STAT3 activation and IL-6 production, leading to tumor growth and metastasis [129]. Thus, inhibiting S1P–S1PR1 signaling and the STAT3 activation pathway may be a useful therapeutic strategy in treating certain cancer types [21,130]. Interestingly in mouse lung tumors, systemic loss of SPHK1 leads to an increase in S1PR1 and a decrease in S1PR2 expression levels [131].

S1PR2 mutational inactivation or deletion confers proliferative advantage in diffuse large B-cell lymphoma (DLBCL) cell lines in vitro and in vivo mouse models [132]. Conversely, the activation of S1PR2, via the (TGF-β)/TGF-βR2/SMAD1 pathway, promotes apoptosis and inhibits DLBCL cell proliferation [132]. Consistently, low S1PR2 expression is associated with worse prognosis, compared to high S1PR2 expression in patients with lymphomas—making S1PR2 a positive prognostic marker for patients with DLBCL [133]. Additionally, ectopic S1PR2 expression decreases lymphoma tumor sizes in vivo [133]. Contrastingly, in endothelial-cell-specific S1pr2 knockout (S1pr2 ECKO) mice, there was a significant decrease of B16F10 melanoma lung metastasis, compared to s1pr2 WT [134]. Consequently, tumors grown in S1pr2 ECKO mice were observed to be smaller, compared to s1pr2 WT [134]. Similarly, targeting SPHK1–S1PR2 signaling reduces acute myeloid leukemia burden and prolonged survival [135]. These data suggest that S1PR2 should be selectively targeted to provide therapeutic options for certain cancer treatments.

In cancer stem cells (CSCs) or tumor-initiating cells, SPHK1 expression enhanced tumor formation via Notch activation stimulated by S1PR3 in both in vitro and in vivo studies [136]. Conversely, tumorigenicity of CSCs was inhibited by knocking down S1PR3 or by using S1PR3 pharmacological antagonists, TY52156 and CAY10444 [136]. In support of these findings, breast cancer patient-derived CSCs were found to contain positive S1PR3/ALDH1 or SPHK1/ALDH1 cells [136]. Specifically in triple-negative breast cancer cell lines, S1P/S1PR3/Notch signaling was found to promote metastasis [137], making S1PR3 a therapeutic target for breast cancer treatment. Additionally, S1PR3 activation was shown to promote cancer progression in osteosarcoma [138] and lung adenocarcinomas [139].

The depletion of S1PR4 was shown to inhibit mammary tumor progression in vivo via CD8^+^ T-cell expansion, since S1PR4 signaling promotes tumor growth by inhibiting CD8^+^ T-cell abundance [140]. This supports the idea that targeting S1PR4 signaling could be a promising strategy to improve anti-CXCR4 cancer immunotherapy [141]. Similarly, lipopolysaccharide was shown to stimulate prostate cancer cell invasion, progression, and metastasis via SPHK1/S1PR4/matriptase signaling [142], indicating that S1PR4 signaling is crucial for the progression of prostate cancer mediated by bacterial infection.

S1PR5, which regulates T-cell infiltration and emigration from peripheral organs [143], was shown to stimulate mitotic progression in HeLa cells, suggesting S1PR5 as a possible therapeutic target for inhibiting tumor proliferation [144]. Specifically, SPNS2–S1P–S1PR5 signaling stimulates the downstream PI3K–AKT–PLK1 pathway that then regulates the metaphase-to-anaphase transition leading to mitotic progression [144].

Collectively, the signaling of all five of the S1P receptors (i.e., S1PR1–S1PR5) has proven to be critical in regulating various kinds of tumor progressions. Thus, selectively targeting these receptors depending on the cancer cell type are potential therapeutic strategies to improve cancer treatment.

### 4.3. Endogenous S1P Signaling Targets

Endogenous S1P generated by either SPHKI or SPHK2 can also act on intracellular targets for signaling functions without needing to engage the S1P receptors or transporters (Figure 4B). In HeLa, HEK 293, and A7 melanoma cells, it was shown that cytoplasmic intracellular S1P generated by SPHK1 is critical for the canonical NF-κB activation pathway by TNF-α—which is necessary for inflammatory immune processes and anti-apoptotic functions [145]. Specifically, endogenous S1P binds TRAF2 (TNF receptor-associated factor 2) at the N-terminal RING domain independent of S1P receptors, leading to lysine-63-linked polyubiquitination of receptor interacting protein 1 (RIP1) and NF-κB signaling activation downstream [145]. Interestingly, TRAF-interacting protein (TRIP) was shown to negatively regulate TNF-induced NF-κB activation by binding to TRAF2 and inhibiting its ubiquitination activity [146]. The TRAF2–TRIP complex formation inhibits the binding of S1P to the TRAF2 RING domain [146]. Contrastingly, in macrophages [147] and keratinocytes [148], intracellular S1P generated by sphingosine kinases was not required for NF-κB activation signaling and inflammation. Furthermore, since SPHK1 and peroxisome proliferator-activated receptor-γ (PPARγ) are known to be expressed in human cancers, PPARγ was reported as a transcription factor target for S1P generated by SPHK1, independent of the S1P receptors [149]. In endothelial cells, S1P binds and activate PPARγ, which then allows for the recruitment of peroxisome proliferator-activated receptor-γ coactivator 1β (PGC1β), forming the SlP/PPARγ/PGC1β complex, to regulate endothelial genes and neoangiogenesis [149]. Consistently, in peripheral T cells, SPHK1-generated S1P binds PPARγ for its transcriptional activation, and the inhibition of SPHK1/S1P/PPARγ signaling ameliorates antitumor immunity against mouse melanoma [19].

Furthermore, SPHK2-derived S1P in the mitochondria was shown to bind homomeric prohibitin 2 (PHB2) with great specificity and affinity without binding to prohibitin 1 (PHB1)—a closely related protein that forms complexes with PHB2 [106,150]. Thus, the S1P–PHB2 complex is critical for mitochondrial respiration functions via cytochrome c oxidase (complex IV) [106]. In the nucleus, SPHK2-derived S1P was reported to bind histone deacetylases (HDACs) 1 and 2 nuclear enzymes, inhibiting histone deacetylation in breast cancer cells [105], thus inducing epigenetic regulation of gene expression [105]. Additionally, SPHK2-generated nuclear S1P was observed to bind directly to human telomerase reverse-transcriptase (hTERT), preventing hTERT from ubiquitination and proteasomal degradation (stabilizing telomerase), leading to enhanced tumor growth [107].

Altogether, both endogenous SPHK1-derived S1P and SPHK2-derived S1P have shown to function independent of their S1P receptor signaling by binding to specific intracellular targets, thereby regulating genes involved in tumor growth/progression.

## 5. Sphingolipid Therapeutics in Cancer

### 5.1. Chemotherapy, Radiotherapy, and Immunotherapy

The combination of sorafenib (multikinase inhibitor) and vorinostat (histone deacetylase inhibitor) was reported to promote CD95 activation by inducing cytosolic Ca^2+^, which increases dihydroceramide levels and reactive oxygen species (ROS), to suppress the growth of gastrointestinal tumor cells and in vivo pancreatic tumors [151]. Consequently, knockdown of CerS6 abolished CD95 activation in tumor cells [151]. Thus, sorafenib plus vorinostat appears to be CerS6-ceramide-dependent, which leads to protein phosphatase 2A (PP2A) and ROS signaling [151,152]. Supportively, the anticancer drug daunorubicin was shown to induce ceramide/ceramide synthase dependent apoptosis in both human leukemia and histiocytic lymphoma cells [65]. However, since ROS production via ASMase/ceramide activation may also induce acute vascular injury [153]; its intracellular production should be controlled in order to prevent long-term side effects in cancer survivors. Additionally, Sorafenib plus vorinostat combination therapy was also shown to improve the efficacy of anti-PD-1 immunotherapy, leading to a significant reduction in pancreatic tumors in vivo [154]. Moreover, gemcitabine (antimetabolite) and doxorubicin (anthracycline) combination therapy was shown to be an effective chemotherapy for some patients with metastatic head and neck cancers [155] via caspase-9/3—dependent mitochondrial cell death by inducing CerS1/C18 ceramide in both in vitro and in vivo xenograft mouse models for head and neck cancers [156]. In the Phase II clinical trials, patients with improved response to gemcitabine plus doxorubicin also had increased in C18 ceramide serum levels [155].

Radiation-induced programmed cell death in *Caenorhabditis elegans* germ cells required ceramide generation via ceramide synthase activation in mitochondria [157]. Moreover, ataxia telangiectasia-mutated (ATM) kinase was shown to regulate the activation of radiation-induced ceramide synthase in the apoptotic response of intestinal crypt clonogen [158]. Interestingly, a neutralizing anti-ceramide monoclonal antibody, which binds ceramide generated for apoptotic signaling, prevented radiation gastrointestinal syndrome mortality in mice [159]. Single-dose radiotherapy/ASMase signaling was shown to ablate more than 90% of human cancers by disabling the homologous recombination of the tumor cells [160,161]. Thus, the induction and regulation of ceramide generation in tumor cells via chemotherapy and radiotherapy are vital therapeutic strategies for cancer treatment.

In addition to chemotherapy and radiotherapy, sphingolipid signaling is also critical in immunotherapy and/or tumor immunology by regulating immune cells for antitumor activities. It was shown that S1PR1 signaling in CD4^+^ T cells promotes breast and melanoma tumor growth via JAK/STAT3 activation in mice, limiting CD8^+^ T-cell recruitment [162]. Additionally, S1P–S1PR1 signaling activates STAT3 by upregulating IL-6 and JAK2 activity to promote tumor growth and metastasis [129]. Consequently, reducing S1P levels by silencing SPHK1 improves the efficacy of anti-CTLA-4 and anti-PD-1 immunotherapy, leading to significant tumor suppression and overall improved survival in mouse melanoma, breast, and colon tumor models [18]. Moreover, patients with Gaucher disease have been shown to have an elevated risk of developing malignant disorders such as multiple myeloma [163]. Interestingly, clonal immunoglobulin in Gaucher disease-associated myeloma patients and mouse models were reactive against lyso-glucosylceramide (due to the fact of a glucocerebrosidase/glucosylceramidase deficiency), which was found to be elevated in both patients and in the mouse models, indicating lyso-glucosylceramide’s involvement in Gaucher disease-associated myeloma origins [164]. Remarkably, activation of complement C5a and C5a receptor 1 (C5aR1) was shown to control glucosylceramide accumulation and inflammatory response in Gaucher disease [165]. Moreover, in a mouse model of leukemia, CerS6-derived C16 ceramide generation was required for optimum T-cell activation and cytokine production in response to alloantigen during allogeneic hematopoietic stem cell transplantation (an effective immunotherapy for hematologic malignances), leading to subsequent graft-versus-host disease (GVHD) induction [166], which is a major complication. However, silencing complement C3aR/C5aR in recipient dendritic cells stimulates lethal mitophagy, owing to ceramide generation and improved GVHD outcome while maintaining the graft-versus-leukemia effect [167]. Collectively, these data show that sphingolipid signaling is crucial in tumor immunology and for the efficacy of immune checkpoint inhibitors in cancer immunotherapy. Surprisingly, understanding the critical roles of sphingolipid signaling in complement biology could be a potential therapeutic strategy for cancer immunotherapy, since the complement components C3/C3a/C3aR and C5/C5a/C5aR signaling are now emerging as potential targets for cancer immunotherapy improvements [168,169,170,171]. Consistently, there have been interesting specific links between bioactive sphingolipids and complement activation in other diseases [172,173,174,175,176].

### 5.2. Anticancer Drugs Targeting Sphingolipids

There are several anticancer drugs that target sphingolipid metabolism or signaling that are being tested for cancer therapy in clinical trials (Table 2).

#### 5.2.1. ABC294640 (Yeliva, Opaganib)

ABC294640, which prevents S1P signaling by selectively inhibiting SPHK2, has been shown to prevent tumor growth via downstream mechanisms involving the inhibition of dihydroceramide desaturase in prostate cancer cells [177], suppression of c-Myc and ribonucleoside-diphosphate reductase subunit M2 (RRM2) in pancreatic cancer cells [178], and the inhibition of telomerase stability in lung cancer cell lines [107]. Additionally, ABC294640 was reported to decrease SPHK2 expression leading to the downregulation of c-Myc and Mcl-1 to induce apoptosis in multiple myeloma [179]. A Phase I study of ABC294640 for patients with advanced solid tumors was successfully completed in which nausea, vomiting, and fatigue were reported as the common drug toxicities [180]. Currently, clinical trials are ongoing for the use of opaganib or ABC294640 to treat patients with metastatic castration-resistant prostate cancer [181] (NCT04207255) and cholangiocarcinoma (NCT03377179)(NCT03414489) (see Table 2).

#### 5.2.2. Fingolimod (FTY720)

The FDA approved drug for multiple sclerosis, FTY720, is a structural analog of naturally occurring sphingosine that acts as a functional antagonist for S1PR1 after its phosphorylation to p-FTY720 by SPHK2 [21,182,183]. Phosphorylated FTY720 internalizes and degrades S1PRs on lymphocytes, thereby depriving them from responding to normal S1P signaling, thus preventing the egression of normal lymphocytes from lymphoid tissues [3]. Because of its mechanism of actions, FTY720 was suggested to be a suitable drug candidate for treating chronic inflammatory-related tumors, as it was shown to prevent colitis-associated cancer (CAC) progression even when given at late stages of disease development [21]. Moreover, in lung cancer [184], multiple myeloma [185], leukemias [186,187,188,189,190,191], and breast cancer stem cells [192], FTY720 mediates cancer cell death and tumor suppression via protein phosphatase 2A (PP2A)-dependent pathways. For instance, in one mechanism, FTY720 induces the formation of large ceramide-enriched membrane pores, called ceramidosomes (ceramide–myosin IIA–RIPK1 complex), leading to necroptosis and lung tumor suppression [193] by directly binding/targeting the oncoprotein I2PP2A/SET, causing PP2A activation [184]. Since FTY720 binds SET, leading to PP2A reactivation, the SET–FTY720 complex was studied using NMR spectroscopy, which revealed that FTY720 binding disrupts SET dimerization, allowing for a specific PP2A trimer activation with tumor suppressive activities [194]. However, an effective use of FTY720 for cancer therapy would be to combine it with a SPHK2 inhibitor in order to prevent the synthesis of p-FTY720, which does not seem to play a role in chronic myeloid leukemia or lung cancer cell deaths [184,188] and also causes immune suppression [8].

#### 5.2.3. Ceramide Nanoliposomes (CNLs)

Ceramide induces programmed cell death in cancer cells, which makes it a potent tumor suppressor [195,196]. However ceramide apoptotic function is also associated with intrinsic toxicities in addition to its poor pharmacokinetics when used alone [197]. Therefore, encapsulating ceramide in nanoliposomes, which forms ceramide nanoliposomes (CNLs), selectively induces cancer cell death while also improving drug solubility and limiting toxic effects [198,199]. C6 ceramide nanoliposomes were shown to prevent tumor growth in hepatocellular cancer mouse models by enhancing their antitumor immune response [200]. Interestingly, targeting survivin with C6 ceramide nanoliposomes induces complete remission of fatal natural killer-large granular lymphocytic (NK-LGL) leukemia in rat models [201]. Moreover, CNL treatment was shown to inhibit metastatic growth in melanoma [202] and ovarian [197] cancer cells, demonstrating that CNL delivery strategy is an effective therapeutic option for cancer treatments.

#### 5.2.4. Sonepcizumab

Sonepcizumab which is a biospecific monoclonal antibody against S1P was shown to prevent tumor progression through S1P neutralization in xenograft and allograft tumor mouse models as well as in in vitro studies using human umbilical vein endothelial cells (HUVECs) [203], making it a promising cancer therapeutic strategy. In a Phase II study, sonepcizumab was assessed in patients with metastatic renal cell carcinoma (mRCC), who had previous history of failed treatments with vascular endothelial growth factor (VEGF) and/or mammalian target of rapamycin (mTOR) inhibitors [204] (NCT01762033). However, the study was later terminated because it did not attain its two-month progression-free survival, which was the primary endpoint [204]. Nevertheless, sonepcizumab had an encouraging overall survival with a median of 21.7 months and a favorable safety profile [204]. Surprisingly, elevated serum S1P levels were observed with sonepcizumab treatment [204], which could explain its limited efficacy in the study, since the active S1P signaling may be blocking antitumor immune response. Therefore, combining sonepcizumab with inhibitors that prevent the synthesis or signaling of systemic S1P could be a much better therapeutic strategy.

There are also other sphingolipid-targeting compounds including safingol, fluphenazine, and desipramine [205] that have been assessed in the clinic for the treatment of different cancer types as shown in Table 2.

## 6. Conclusions and Future Directions

Sphingolipids, as complex biological molecules, have been shown to regulate various biological/cellular processes including tumor cell death and survival. The cloning of key sphingolipid enzymes has helped in understanding the mechanisms and functions of sphingolipids, such as ceramides and S1P, in regulating cancer signaling. Ceramide has emerged as a tumor suppressor by facilitating necroptosis, mitophagy, apoptosis, and lethal autophagy [74,206]. Development of compounds that lead to ceramide synthesis has proven to be a novel anticancer therapeutic strategy. However, the accumulation of ceramides also comes with intrinsic toxicities, which makes the use of CNL an effective therapeutic strategy for ceramide-based drug delivery.

S1P, which is known for its pro-tumorigenic effects and inducing tumor progression, has also emerged as a promising target for cancer treatment with few ongoing clinical trials. Sonepcizumab, the monoclonal antibody against S1P that inhibits tumor growth in xenograft models [203], was not an effective S1P blockade in clinical trials for solid tumors [204], emphasizing the need for combinatorial therapies for sphingolipid-based drugs in developing an effective anticancer treatment.

Analysis from the TCGA cancer patient panels revealed that sphingolipid metabolic enzymes are dysregulated with heterogeneity in various cancer types, which are dependent on context and cell type. For instance, although it was initially reported that PF-543 (a potent SPHK1 selective inhibitor) significantly decreased endogenous S1P levels by 10-fold in MD-1483 head and neck carcinoma cells, the data showed no effects on proliferation or survival [207]. However, recent studies show that PF-543 inhibits tumor growth in colorectal cancer cell lines and in xenograft mouse models [208]. Going forward, it is particularly important to define which cell type produces which sphingolipid enzyme in a specific cancer type, which will help in the development of effective future sphingolipid-based anticancer therapeutics.

Finally, to effectively develop sphingolipid based therapeutics for cancer treatment, we must continue to elucidate and understand specific mechanisms of sphingolipids in regulating cancer signaling. Additionally, effective sphingolipid-based drugs against cancers should be based on a combinatorial therapeutic regimen targeting different pathways to maintain sphingolipid metabolism homeostasis and avoid toxic side effects or drug resistance.

## Figures and Tables

**Table 1:** Key Sphingolipid enzymes and their roles in cancer progression.

Enzymes	Metabolic Functions	Roles in Cancer	References
SPHK1	S1P generation	Promotes tumor growth in melanoma, ovarian, and colitis-associated cancers	[18,19,20,21,22,23]
SPHK2	S1P generation	Augments 5-FU chemotherapy resistance in human colorectal cancer and mediates FSH-induced cell proliferation in ovarian cancer	[22,23]
SGPL1	Irreversibly breaks down S1P	Inhibits colon tumor formation and prevents S1P-induced migration and cell-colony formation in pediatric alveolar rhabdomyosarcoma	[24,25,26,27]
CERK	C1P generation	Promotes breast cancer growth and confers chemotherapy resistance to breast cancer cell lines	[31,32,33,34]
AC	Cleaves fatty acid moiety from ceramide	Overexpressed in several cancer types and mediates the switch between proliferative and invasive phenotype states in melanoma cells. It also confers resistance to cancer cell death	[36,37,38,39,40,41,42]
NC	Cleaves fatty acid moiety from ceramide	Inhibits cellular apoptosis in colon cancer cells and induces xenograft tumor growth	[44,45]
ACER3	Cleaves fatty acid moiety from ceramide	Promotes tumor growth and inhibits apoptosis in HCC and AML cells	[57,58]
CerS1	Synthesis of C18 ceramide	Inhibits HNSCC xenograft growth and induces cancer cell death	[69,70,71,72,73]
CerS2	Synthesizes very-long-chain ceramides	Inhibits in vivo metastasis and invasiveness of ovarian cancer cells	[75]
		Partially prevents programmed cell death induced by ionizing radiation in HeLa cells	[77]
CerS4	Synthesis of C18–C20 ceramides	Inhibits A549 cancer cell migration and invasion	[79]
CerS6	Generates C16 ceramide	Promotes cell proliferation in PDAC, HNSCC, and lung cancer cell lines	[71,83,84]
		Has anti-proliferative and pro-apoptotic functions in polyploid giant cancer cells	[85]
Alk-SMase (ENPP7)	Ceramide generation	Reduces colon cancer progression in a mice model	[87]
ASMase (SMPD1)	Ceramide generation	ASMase’s induction in platelets induces B16F10 melanoma metastasis	[89,90,91]
NSMase2 (SMPD3)	Ceramide generation	Enhances the efficacy of anti-PD-1 antibody therapy in melanoma and breast cancer mouse models. It also inhibits tumor progression in oral squamous cell carcinoma	[92,93]
SMS2 (SGMS2)	Produces sphingomyelin and diacylglycerol	Promotes ovarian cancer cell growth and migration	[99]

SPHK1, sphingosine kinase 1; SPHK2, sphingosine kinase 2; SGPL1, sphingosine-1-phosphate lyase 1; CERK, ceramide kinase; AC, acid ceramidase; NC, neutral ceramidase; ACER3, alkaline ceramidase 3; CerS1, ceramide synthase 1; CerS2, ceramide synthase 2; CerS4, ceramide synthase 4; CerS6, ceramide synthase 6; Alk-SMase (ENPP7), alkaline sphingomyelinase; ASMase (SMPD1), acid sphingomyelinase; NSMase2 (SMPD3), neutral Sphingomyelinase; SMS2 (SGMS2), sphingomyelin synthase 2; S1P, sphingosine-1-phosphate; 5-FU, 5-fluorouracil; FSH, follicle-stimulating hormone; C1P, ceramide-1-phosphate; HCC, hepatocellular carcinoma; AML, acute myeloid leukemia; HNSCC, head and neck squamous cell carcinoma; PDAC, pancreatic ductal carcinoma.

**Table 2:** Clinical trial drugs targeting sphingolipid metabolism for cancer treatments.

Name	Sphingolipid Targets	Cancer Type	Stage	ClinicalTrials.gov Identifier
ABC294640 (Yeliva, opaganib)	SPHK2; DES	Prostate Cancer	Phase II	NCT04207255
		Multiple Myeloma	Phases I and II	NCT02757326
		Cholangiocarcinoma	Phase II	NCT03377179, NCT03414489
Fingolimod (FTY720) (FDA approved for MS)	S1PR1	Breast Carcinoma (treating paclitaxel-associated neuropathy)	Phase I	NCT03941743
		Glioblastoma & Anaplastic Astrocytoma (treating severe and persistent lymphopenia in patients undergoing radiation and chemotherapy)	Early Phase I	NCT02490930
ASONEP™ (sonepcizumab/LT1009)	S1P	Solid Tumors	Phase I	NCT00661414
Ceramide NanoLiposome	Ceramide inducer	Renal Cell Carcinoma	Phase II	NCT01762033
		Solid Tumors	Phase I	NCT02834611
		Acute Myeloid Leukemia	Phase I	NCT04716452
Safingol	SPHK1	Locally Advanced or Metastatic Solid Tumors	Phase I	NCT00084812
Fluphenazine	ASMase	Multiple Myeloma and Plasma Cell Neoplasm	Phases I and II	NCT00335647
		Multiple Myeloma	Phase I	NCT00821301
Desipramine	AC	Small Cell Lung Cancer and Neuroendocrine Tumors	Phase II	NCT01719861

AC, acid ceramidase; DES, dihydroceramide desaturase; S1P, sphingosine-1-phosphate; S1PR, S1P receptor; SPHK, sphingosine kinase; ASMase, acid sphingomyelinase; FDA, The United States Food and Drug Administration; MS, multiple sclerosis.
